# Extracellular Vesicle-Contained microRNA of *C. elegans* as a Tool to Decipher the Molecular Basis of Nematode Parasitism

**DOI:** 10.3389/fcimb.2020.00217

**Published:** 2020-05-25

**Authors:** Thomas B. Duguet, Julien Soichot, Rostyslav Kuzyakiv, Lars Malmström, Lucienne Tritten

**Affiliations:** ^1^Institute of Parasitology, McGill University, Sainte-Anne-de-Bellevue, QC, Canada; ^2^Institute of Parasitology, University of Zurich, Zurich, Switzerland; ^3^S3IT, University of Zurich, Zurich, Switzerland; ^4^SIB Swiss Institute of Bioinformatics, Lausanne, Switzerland; ^5^Institute for Computational Science, University of Zurich, Zurich, Switzerland; ^6^Division of Infection Medicine, Lund University, Lund, Sweden

**Keywords:** nematode, host, microRNA, secreted, extracellular vesicles, computational analysis, immunomodulation, host-parasite relationships

## Abstract

Among the fundamental biological processes affected by microRNAs, small regulators of gene expression, a potential role in host-parasite communication is intriguing. We compared the miRNA complement of extracellular vesicles released by the free-living nematode *Caenorhabditis elegans* in culture to that of other adult parasitic nematodes. Expecting convergent functional roles for secreted miRNAs due to the common parasitic lifestyle of the organisms under investigation, we performed a miRNA sequence analysis as well as target search and pathway enrichment for potential mRNA targets within host immune functions. We found that the parasite miRNA seed sequences were more often identical to those of *C. elegans*, rather than to those of their hosts. However, we observed that the nematode-secreted miRNA fractions shared more often seed sequences with host miRNAs than those that are not found in the extracellular environment. Development and proliferation of immune cells was predicted to be affected several-fold by nematode miRNA release. In addition, we identified the AGE-RAGE signaling as a convergent targeted pathway by species-specific miRNAs from several parasitic species. We propose a multi-species comparative approach to differentiate those miRNAs that may have critical functions in host modulation, from those that may not. With our simple analysis, we put forward a workflow to study traits of parasitism at the miRNA level. This work will find even more resonance and significance, as an increasing amount of parasite miRNA collections are expected to be produced in the future.

## Introduction

MicroRNAs (miRNAs) represent a class of non-coding RNAs that function as regulators of gene expression, impacting multiple and various physiological functions within eukaryotic organisms. Approximately 20 nucleotides in length, these small molecules target messenger RNA (mRNA) by complementary base pairing and induce mRNA degradation with subsequent inhibition of translation (Bartel, 2009; Fabian et al., 2010). This phenomenon was first described in the free-living nematode *Caenorhabditis elegans* (Lee et al., 1993; Wightman et al., 1993; Reinhart et al., 2000), which led to the subsequent identification of so far over 2,500 human miRNAs predicted to target at least 60% of human genes (Lewis et al., 2005; Friedman et al., 2009). The past decade has seen numerous reports of the ubiquitous nature of miRNA-derived regulation of gene expression while enriching current databases with 38,589 miRNAs in the most recent miRBase release (v. 22) (Griffiths-Jones et al., 2008; Kozomara et al., 2019).

Among the fundamental biological processes affected by miRNAs, a potential role at the host-parasite interface is intriguing. Host-parasite interactions, shaped by thousands of years of co-evolution, are characterized by a highly complex molecular dialogue, including the release and transport of miRNAs. Recent studies suggest that parasites utilize their secreted miRNAome to divert and modulate responses of the host immune system to favor their own development (reviewed in Eichenberger et al., 2018b; Tritten and Geary, 2018). These interactions are at least partly mediated through the secretion and trafficking of parasite-derived extracellular vesicles (EVs), which transport a large diversity of bioactive proteins, lipids, and nucleic acids, among which miRNAs are highly represented (Valadi et al., 2007; Bernal et al., 2014; Buck et al., 2014; Eichenberger et al., 2018a; Tritten and Geary, 2018).

The significant level of identity between host and parasite miRNAs, including for *Brugia malayi* and *Fasciola hepatica* and their respective hosts, was emphasized (Buck et al., 2014; Fromm et al., 2015; Zamanian et al., 2015). This putative convergent evolution is predicted to endow the parasite with the capacity to hijack host miRNA regulatory networks. This identity extends to entire parasite-derived miRNA sequences, but may also be critically important even if restricted to the seed region that binds to cognate mRNAs.

The nematode *C. elegans* has been extensively used as a model organism for research in developmental biology. Its genome was the first to be sequenced and its miRNAome is very well-characterized, with 253 precursors and 437 mature sequences in miRBase release 22 (Kozomara et al., 2019). Based on its free-living lifestyle and our extensive knowledge of its genome and biology, comparing nematodes such as *C. elegans* with parasitic nematodes can be used to uncover the molecular basis of parasitism. *C. elegans*, too, release EVs, which play crucial roles in development and behavior (Beer and Wehman, 2017). The model has also proved valuable to improve our mechanistic understanding of EV release, and the components involved in EV formation (Wehman et al., 2011; Hyenne et al., 2015; Beer and Wehman, 2017).

To date, the secreted miRNAomes of many nematode species have been sequenced and characterized, providing a basis for a computational and experimental effort to identify the targets of key immunomodulatory parasite-derived miRNAs. Here, we have characterized the miRNA complement of EVs released by *C. elegans* in culture. We compared our data to the excretory/secretory (E/S) miRNAs released by other adult parasitic nematodes *in vitro*. Expecting convergent functional roles for secreted miRNAs due to the common parasitic lifestyle of the organisms under investigation, we performed target search and pathway analysis for potential mRNA targets within host immune functions.

## Materials and Methods

### *C. elegans* Cultures and Media Processing

The hermaphrodite strain N2 (Bristol) was obtained from the *Caenorhabditis* research center, University of Minnesota. They were fed with *Escherichia coli* OP50, maintained, and synchronized as described (Stiernagle, 2006). Synchronized young adult cultures were obtained 3 days after seeding ~10,000 eggs per nematode growth medium plate. Young adults were thoroughly washed before incubations for 5 or 24 h in M9 buffer (Stiernagle, 2006). Between 35,500 and 158,000 worms were used for each experiment (listed in Supplementary Table S1). Worms were removed from media by sedimentation and supernatants were processed by sequential centrifugations at 4°C: 10 min at 1,500 × g and 45 min at 12,000 × g. Ultracentrifugations were performed in a Beckman ultracentrifuge (Optima L-80 XP), using a SW40Ti rotor and Ultra-Clear thinwall tubes (14 × 95 mm, Beckman 344060) as follows: 2 h at 120,000 × g, 4°C; the pellet was washed with cold sterile PBS (Gibco), and centrifuged again for 2 h at 120,000 × g, 4°C, as described (Théry et al., 2006; Sotillo et al., 2016). The resulting pellet was lyzed in Trizol reagent (Ambion) and stored at −20°C until further processing. Total RNA was isolated from all 4 samples in parallel, using a modified phenol/chloroform extraction protocol designed to increase the yield and quality of small RNA species (Zununi Vahed et al., 2016). Briefly, following the Trizol reagent manufacturer's instructions and after the usual phase separation, 3M potassium acetate pH 5.2 was added at 1:10 ratio to the collected aqueous phase, incubated for 30 min at −20°C, and spun at 12,000 × g for 12 min at 4°C. Subsequently, the supernatant was mixed with 1 equal volume of 2.5 M LiCl and 2 equal volumes of pre-chilled ethanol. After 2 h at −80°C, the mixture was spun at 16,000 × g for 20 min at 4°C. Pellets were air-dried, solubilized in nuclease-free dH_2_O, and stored at −80°C. RNA from two 5 and 24 h incubations each, respectively, were pooled and shipped on dry ice to LC Sciences for miRNA sequencing.

### miRNA Sequencing and Analysis

RNA quality was checked using an Agilent Technologies 2100 Bioanalyzer (Supplementary File S1). The library was prepared following Illumina's TruSeq small-RNA-sample preparation protocols. Single-end sequencing (50 bp) was performed on Illumina's HiSeq 2500 sequencing system. Raw reads were subjected to LC Sciences proprietary program, ACGT101-miR (LC Sciences, Houston, Texas, USA) to analyze the data. Adapter dimers, junk, low complexity, common RNA families (rRNA, tRNA, snRNA, snoRNA) and repeats were removed (Supplementary File S1). Unique sequences with 18~26 nucleotides in length were mapped to *C. elegans* precursors in other nematode miRNA precursors in miRBase v. 22 to identify known miRNAs and novel 3p- and 5p- derived miRNAs. The unmapped sequences were BLASTed against the *C. elegans* genome, and hairpin structures were predicted from the flanking 80 nt sequences using RNAfold (http://rna.tbi.univie.ac.at/cgi-bin/RNAWebSuite/RNAfold.cgi). Normalization of sequence counts in each sample was achieved by dividing the counts by a library size parameter of the corresponding sample, as in Tritten et al. (2016; GEO accession GSE144289). Mature sequences were re-annotated using MirGeneDB 2.0, a database containing recently curated miRNA information (Fromm et al., 2020).

### Mass Spectrometry Analysis

EV-enriched samples from two independent incubations of 5 and 24 h each were submitted for mass spectrometric analysis at the Functional Genomics Center Zurich. Briefly, samples were precipitated using trichloroacetic acid. Protein pellets were washed twice with cold acetone, dried and resuspended in 45 μl 10 mM Tris, 2 mM CaCl_2_, pH = 8.2, and 5 μl trypsin 100 ng/μl in 10 mM HCl). A microwave assisted digestion of 30 min at 60°C was observed, after which samples were dried and dissolved in 10 μl ddH_2_0 + 0.1% formic acid. Protein concentrations were measured by Nanodrop. For the LC-MS/MS analysis, 105 and 195 ng (5 h samples), and 212 and 420 ng (24 h samples) digested protein were injected, respectively, on a nanoAcquity UPLC coupled to a Q-Exactive mass spectrometer (Thermo Scientific). Database searches were performed on a Mascot search engine Perkins et al. (1999) against the *Escherichia coli* and *C. elegans* databases (downloaded from Uniprot on 19 November 2019 and from Wormbase on 18 November 2019, respectively), independently and sequentially, in order to maximize number of hits. Further analysis was performed in Scaffold v. 4 (Proteome Software) using stringent settings: 1% protein FDR, a minimum of 2 peptides per protein, and 0.1% peptide FDR. In Scaffold, a quantitative analysis was performed based on normalized total spectra (default parameters) whereby the sum of all the spectra associated with a specific protein within a sample is used, which includes also those spectra that are shared with other proteins and is referred to as the total spectrum count. Proteins were further analyzed by gene ontology using Blast2GO (Götz et al., 2008).

### Transmission Electron Microscopy and Nanoparticle Tracking Analysis

Representative *C. elegans* supernatants processed as above (twice independently) were used to verify the presence of extracellular vesicles by transmission electron microscopy at the center for microscopy and imaging analysis (University of Zurich). For negative staining, samples were fixed by adding 4% formic acid in PBS at a 1:1 ratio with sample. Samples were glow-discharged for 20 min on the grid, washed with PBS, and 1% glutaraldehyde was added for 5 min. Samples were washed 5 × 2 min in dH_2_O. A contrast enhancement step of 5 min with uranyl acetate 1% was performed, followed by 10 min incubation on ice with uranyl acetate/methylcellulose (900 μl methylcellulose 2%/100 μl 3% uranyl acetate). Samples were dried and imaged by transmission electron microscopy (TEM). Nanoparticle tracking analysis (NTA; ZetaView, Particle Metrix, Germany) was performed on a fresh 24 h incubation sample, diluted 1:250 in commercial PBS (Gibco) confirmed to be particle-free by NTA. Measurements of particle concentration and size were based on 11 frames.

### Comparative Analysis Across Several Nematode Species

Experimental E/S miRNA collections from *in vitro* studies on adult gastrointestinal nematodes were retrieved from the literature, for which sequence data was available. These comprised miRNAs from *Ascaris suum* (*n* = 29; Hansen et al., 2019), *Haemonchus contortus* (*n* = 40; Gu et al., 2017), and *Trichuris muris* (*n* = 56; Eichenberger et al., 2018c). miRNA family annotations were obtained from MiRGeneDB 2.0 (Fromm et al., 2020), based on homology with *C. elegans* and *A. suum* mature miRNA collections and/or miRBase v. 22 (Kozomara et al., 2019). High-confidence E/S miRNA data from *H. polygyrus*, present in 10 copies or more in the worm secretions (although not confined to EVs) and registered in miRBase v. 22, were included in the seed sequence identity analysis (*n* = 74; Buck et al., 2014).

UpSetR package was used to plot the extent of common and unique seed sequences (nucleotides 2–8) across experimentally validated E/S parasite miRNA datasets (Lex et al., 2014).

miRNAs from the corresponding mammalian host species were also extracted from miRBase (v. 22): *Sus scrofa* (*n* = 457), *Mus musculus* (*n* = 1,978), *Ovis aries* (*n* = 153), as well as those from *C. elegans* (*n* = 437). In order to evaluate seed sequence conservation toward host miRNAs vs. nematode miRNAs, we assessed the proportion of identical seed sequences in parasite vs. host, and in parasite vs. *C. elegans* in a similarity matrix (Hamming distance), as proposed previously (Kehl et al., 2017). Briefly, the matrices consisted of pairwise comparisons between (i) all parasite and host seeds for each host-parasite association, and (ii) all parasite vs. *C. elegans* seeds (using 437 *C. elegans* mature miRNAs from miRBase). Approximate string distances were calculated for each nematode/host miRNA pair with the function “stringdist” package (van der Loo, 2014) in R v. 3.5.1 (method = hamming). The number of nucleotides matching in position between two sequences was used to create graphs expressed as a percentage of the total number of comparisons. Whether the proportions of 100% matching seeds between parasite vs. host and parasite vs. *C. elegans* are equal was tested in two-proportions z-tests (two-sample test for equality of proportions with continuity correction) in R v. 3.5.1. Briefly, we tested whether the observed proportion of identical nematode seeds against host miRNAs is greater than the observed proportion of nematode seeds against *C. elegans* miRNAs.

miRNA target prediction was performed using the TargetScan software package v. 7 (http://www.targetscan.org), implemented as a standalone workflow under iPortal (Kunszt et al., 2015) and openBIS (Bauch et al., 2011), using default parameters (Agarwal et al., 2015). The 84 species alignment allows mapping by homology. All host genes with a weighted context ++ score ≤ −1.0 and targeted at least twice were used in enrichment analyses, as described (Benna et al., 2019). The R version of the program Enrichr was employed, using the “KEGG_2019_Mouse,” and “Reactome_2016” databases (Kuleshov et al., 2016).

## Results

### EVs Found in the Secretions of *C. elegans* Contain Numerous Proteins and miRNAs

Imaging by transmission electron microscopy (TEM) revealed a heterogeneous EV population in our samples, as shown in Supplementary Figure S1. Some EVs show the typical deflated ball structure as well as the size of exosomes (about 100 nm Ø). Further smaller (possibly) vesicular structures of ~20 nm, which may represent *E. coli* EVs. Nanoparticle tracking analysis (NTA) of a representative sample (24 h incubation) revealed a median particle size of 107.3 nm Ø (Supplementary Figure S2) and a concentration of 1.5 × 10^10^ particles per ml.

Protein species associated with the EV-enriched fractions of culture supernatants were identified by mass spectrometry. The large majority of identified proteins originated from *E. coli*, with 753 proteins (Supplementary Table S2). A total of 170 unique proteins (based on total unique peptide counts, all samples confounded) were identified as *C. elegans* proteins; 128 were observed in the secretions after both 24 and 5 h incubations, 62 were only seen after 24 h incubation and 37 were only detected after 5 h of incubation (Supplementary Table S2). Based on our stringent search criteria, one sample (5 h_2) displayed a different and less diverse protein profile (only 51 proteins) compared to the three others, which were in agreement regarding the ranking in abundance, for unexplained reasons. In each of these three samples, eight proteins were common to the top 10 with slight variations in the exact ranking. These included two IRG-7 proteins (Q19853 and A0A131MBU3), two members of the neprilysin metallopeptidase family (B6VQ96 and Q22763), two carboxypeptidases (Q94269 and K8ESM2), two uncharacterized proteins with a C-type lectin domain (G5ECR0), and regulated by DAF-16 (H2L0Q1). The outlying 5 h sample showed one uncharacterized common protein with the other samples' top 10 (H2L0Q1), two actins (P10986 and O45815), two lysozymes (O62415 and Q20964), as well as four proteins with protease/peptidase activity (P34528, Q94271, Q22972, and O01530). Actin was detected in both 5 h samples but not after 24 h. Highly represented in the *C. elegans* EV-enriched secretions were proteins with protease/(carboxy)peptidase activity, and carbohydrate binding properties. In line with the experimental design, 80/127 (63%) proteins assigned to a gene ontology cellular component (74% of all proteins) were associated with membrane (GO:0016020, GO:0016021, GO:0005886, GO:0045121), or the extracellular space (GO:0005615, GO:0005576). This is in agreement with a previous report (Russell et al., 2018).

miRNA sequencing revealed 100 high confidence miRNA species in our samples in total. These encompassed miRNAs from groups 1, and 2a, present in 10 copies or more (see Supplementary Table S3). This represents ~20% of the known *C. elegans* mature miRNAs registered in miRBase. We found 94 and 69 high confidence miRNA species in samples resulting from 5 and 24 h incubation, respectively. Table 1 shows the most abundant high confidence miRNAs found in each sample, based on normalized read counts. The agreement between the two samples was good: 11/15 most abundant miRNAs at 5 h were also among the top 15 after 24 h incubation.

**Table 1:** Top 15 most abundant high confidence mature miRNAs in the supernatants of *C. elegans* after 5 h and 24 h in liquid culture.

**Abundance rank**	***C. elegans* 5 h**	***C. elegans* 24 h**
1	**cel-bantam-P2_3p (cel-miR-80-3p)**	**cel-let-7-P5_5p (cel-let-7-5p_1ss12GA)**
2	**cel-miR-54-P3_3p (cel-miR-56-3p)**	**cel-miR-54-P3_3p (cel-miR-56-3p)**
3	**cel-miR-36-P1_3p (cel-miR-35-3p)**	**cel-bantam-P2_3p (cel-miR-80-3p)**
4	**cel-let-7-P5_5p (cel-let-7-5p_1ss12GA)**	**cel-miR-36-P1_3p (cel-miR-35-3p)**
5	**cel-miR-52-P1_5p (cel-miR-52-5p)**	**cel-miR-10-P2l_5p (cel-miR-51-5p)**
6	**cel-miR-10-P2l_5p (cel-miR-51-5p)**	**cel-bantam-P4_3p (cel-miR-82-3p)**
7	**cel-miR-36-P3_3p (cel-miR-37-3p)**	**cel-miR-54-P2_3p (cel-miR-55-3p)**
8	**cel-miR-10-P3e_5p (cel-lin-4-5p)**	**cel-miR-36-P3_3p (cel-miR-37-3p)**
9	**cel-miR-96-P3_5p (cel-miR-228-5p)**	**cel-miR-52-P1_5p (cel-miR-52-5p)**
10	**cel-miR-36-P6_3p (cel-miR-40-3p)**	cel-miR-90_3p (cel-miR-90-3p)
11	**cel-miR-54-P2_3p (cel-miR-55-3p)**	**cel-miR-36-P6_3p (cel-miR-40-3p)**
12	cel-miR-10-P3f_5p (cel-miR-237-5p)	cel-miR-10_3p (cel-miR-238-3p)
13	cel-miR-29-P2_3p (cel-miR-49-3p)	cel-miR-1_3p (ppc-miR-1_R+2)
14	**cel-bantam-P4_3p (cel-miR-82-3p)**	**cel-miR-96-P3_5p (cel-miR-228-5p)**
15	cel-miR-31_5p (cel-miR-72-5p_R-2)	**cel-miR-10-P3e_5p (cel-lin-4-5p)**

*cel, C. elegans; ppc, Pristionchus pacificus. Both annotations provided by MirGeneDB and miRBase (in brackets) are shown. Deviations from sequences registered in miRBase are indicated as follows: ss, substitutions; R, addition or deletion of nucleotides on the 3′ end of the mature sequence. Names indicated in bold show miRNA candidates common to the top 15 of both incubation periods. Please note that all sequences listed in this table are present in material from both 5 and 24 h*.

### *C. elegans* vs. Other Nematode miRNAs

**Figure 1:**
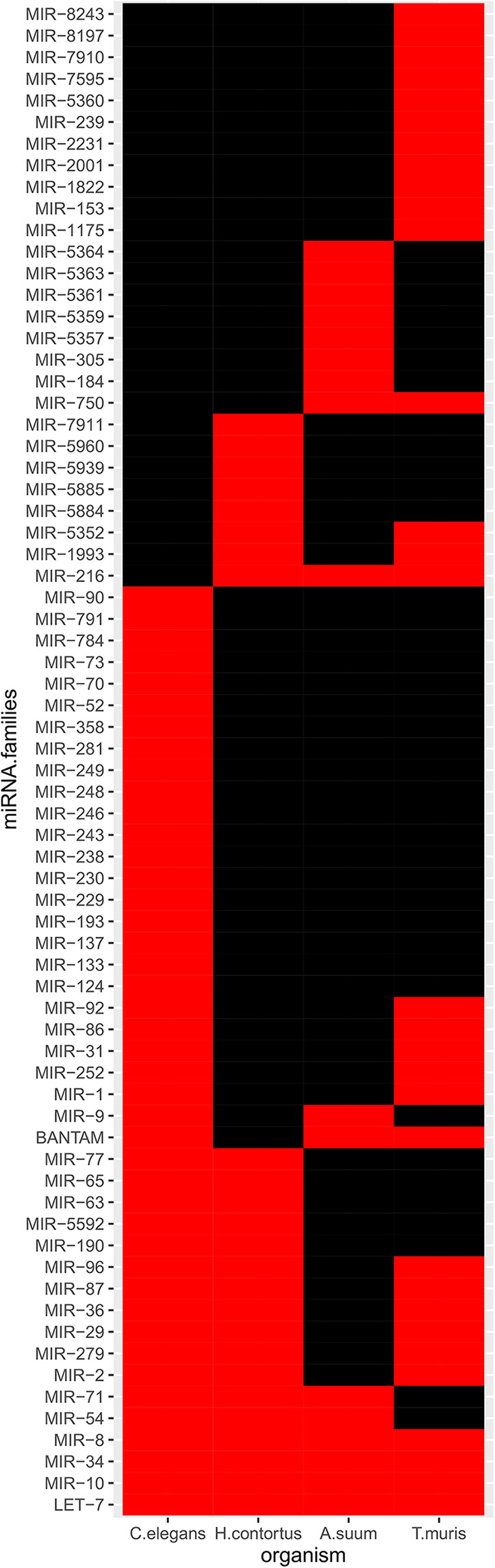
miRNA families in available EV-contained datasets. Only high-confidence *C. elegans* miRNAs were considered. Black, absence; red, presence.

Experimental EV-contained miRNA collections from *in vitro* studies on adult gastrointestinal nematodes were retrieved from the literature, for which sequence data was available. These comprised miRNAs from *A. suum, H. contortus*, and *T. muris*. Based on MirGeneDB family annotations, the LET-7, MIR-10, MIR-34, and MIR-8 (miR-236 in miRBase) families were consistently identified across all datasets, including among the high-confidence *C. elegans* miRNAs (Figure 1 and Supplementary Table S4). The MIR-216 family (hco-miR-259-5p, asu-miR-5362-5p, asu-miR-5342-3p according to miRBase) was only represented in parasitic species, in the three species examined. The MIR-71 and MIR-54 families were detected in the EVs from all species except *T. muris*. Similarly, the BANTAM family was represented in EVs from all except *H. contortus*. The MIR-2, MIR-36, MIR-29, MIR-279, and MIR-87 families were not described in EVs from *A. suum*.

**Figure 2:**
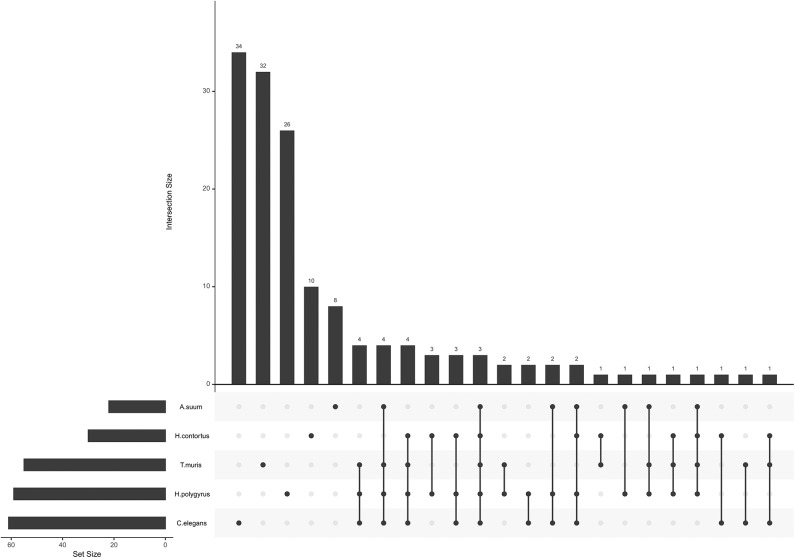
miRNA seed sequences across secreted nematode datasets. The plot was generated with all unique seed sequences across each dataset. The intersection size represents the number of unique seed sequences; (connected) dots under the bar chart indicate the distribution across datasets and show sequences shared by multiple datasets.

miRNA seed sequences mediate most of the interaction with and confers specificity to the target mRNA. Unique seed sequences (i.e., a given seed appears only once in the list, even if common to several miRNAs) were compared across datasets, this time also including *H. polygyrus* miRNAs (E/S miRNAs not restricted to EVs; Figure 2). Most seed sequences appeared to be unique to each miRNA collection; three seed sequences were shared by all five species among secreted datasets and using miRBase annotations [ACCCGUA, as in miR-100 (or miR-51-56 in *C. elegans*); GAGAUCA, as in miR-81, and GAGGUAG, as in let-7]. One sequence was common to the four parasites, but absent from *C. elegans* (AAGCUCG; as in miR-993). A given seed sequence may not stem from the same miRNA, but may target the same genes. Based on the limited number of studies and species examined here, there is *a priori* no clade-clustering with our small study sample, besides three seeds shared by clade V organisms (*H. contortus, H. polygyrus, C. elegans*; GAUAUGU, as in miR-50; UCAUCAG, as in miR-5899, and AUGACAC, as in miR-60,−63, -64).

The mRNA targeting properties of miRNAs is directly related to the seed sequence. Here, we focused on the nucleotides in position 2–8 on the mature sequence. Considering the possibility of a potential evolutionary advantage for the parasite to secrete miRNAs sharing identical seeds with its host's and so, subvert to existing host regulatory pathways, we hypothesized that a higher proportion of seed sequence identity (7/7 matching nucleotides) would be observed compared to host miRNAs than to *C. elegans* miRNAs. To test this hypothesis, nematode parasite E/S miRNA seeds (nucleotides 2–8) were aligned to all host miRNAs, creating similarity matrices. The same was done with non-E/S miRNA helminth seeds, aligned to host or *C. elegans* miRNA seeds. Systematic pairwise comparisons between all parasite and host or *C. elegans* miRNAs resulted in between 5,814 and 816,501 comparisons. This is due to the varying numbers of miRNAs available from miRBase for each host species, and to the number of nematode miRNA entries. Applying a two-sample test for equality of proportions with continuity correction, the hypothesis was refuted for every nematode-host association (Table 2). On the contrary, nematode E/S miRNAs were more conserved (i.e., more often identical) toward the phylogenetically closer organism, since the proportion of identical seeds was higher in alignments to *C. elegans* than to host miRNA seeds (*p* < 0.05). However, nematode E/S miRNA seeds were found to be more often identical to host miRNAs than non-E/S (somatic) miRNAs (*p* < 0.001). This was also the case for *C. elegans* artificially aligned to mouse miRNAs. Similarly, parasite E/S miRNA seeds were also found to be more often identical to *C. elegans* miRNAs than non-E/S miRNA seeds (*p* < 0.001). This suggests that miRNA species that tend to be secreted may be more conserved across nematodes, than somatic miRNAs.

**Table 2:** Seed identity across miRNAs from nematodes, hosts, and *C. elegans*.

	**Parasite vs. host (in % alignments)**		**Parasite vs**. ***C. elegans*** **(in % alignments)**		**Are nematode E/S miRNA seeds more often identical to their host's miRNAs than to those of *C. elegans*?**	**Are nematode E/S miRNA seeds more often identical to their host's miRNAs than non-E/S miRNAs?**
	**E/S**	**Non-E/S**	**E/S**	**Non-E/S**		
**Proportion of identical seeds across repeated pairwise alignments**						
C. elegans^*^	0.09	0.02	NA	NA	NA	Yes (*p* < 0.001)
*H. contortus*	0.34	0.08	0.61	0.14	No (*p* = 0.99)	Yes (*p* < 0.001)
*H. polygyrus*	0.07	0.01	0.14	0.09	No (*p* = 1)	Yes (*p* < 0.001)
*A. suum*	0.36	0.06	0.59	0.28	No (*p* = 0.99)	Yes (*p* < 0.001)
*T. muris*	0.04	NA	0.24	NA	No (*p* = 1)	NA

*Each nematode miRNA seed set (nt 2–8, E/S, and non-E/S) was aligned pairwise to each host or C. elegans total miRNA list from miRBase. This resulted in thousands of alignments, of which a proportion showed 100% match (7/7 nucleotides)*.

* *C. elegans miRNAs were aligned to mouse miRNAs, which is the largest host miRNA dataset*.

### Predicted Targets in Host Genomes

Host genes predicted to be targeted by helminth miRNAs were used in pathway enrichment analyses. The number of genes resulting from target predictions from each helminth-host association and used for downstream analysis was as follows: *A. suum*/*S. scrofa* (*n* = 3,594), *H. contortus*/*O. aries* (*n* = 3,833), *T. muris*/*M. musculus* (*n* = 13,952), *H. polygyrus*/*M. musculus* (*n* = 14,525), *C. elegans*/*M. musculus* (*n* = 15,111), *C. elegans*/*S. scrofa* (*n* = 9,896), *C. elegans*/*O. aries* (*n* = 8,510). Interrogating the KEGG and Reactome databases via the tool Enrichr returned hundreds of overrepresented pathways. Significantly overrepresented pathways (adjusted *p* < 0.05) were compared across helminth-host associations including *C. elegans* and the various mammalian hosts used in this study (Table 3 and Supplementary Table S5). While no KEGG term was uniquely associated with parasitism (i.e., present in all parasitic species, but absent in the *C. elegans* vs. hosts predictions), 34 terms were common to all seven associations. These included T cell receptor signaling pathway, as well the interconnected Ras, Rap1, FoxO, and ErbB signaling pathways. Similarly, the putative miRNA targets of helminth miRNAs were enriched in genes belonging to 18 Reactome pathways (adj. *p* < 0.05) common to all helminth-host associations. Among those, 12 pathways (with extensive redundancy) are related to the PI3K/Akt signaling (not shown). Again, this qualitative analysis did not allow the clustering of pathways associated with parasitic lifestyle. Pathway analysis further revealed some features of interest among host-parasite associations (without *C. elegans*), worthy of attention. For instance, mucin synthesis is predicted to be affected in the pig by *A. suum* miRNAs, which was not observed in the other host-parasite pairs. Notch signaling pathway is targeted by *A. suum* and *H. polygyrus* miRNAs, while TGF-β signaling counted among targeted pathways by *H. contortus, T. muris*, and *H. polygyrus* miRNAs in their respective hosts. The mTOR signaling pathway might be altered by all four parasites, while the capacity of Th17 cell differentiation may be modulated by *A. suum* miRNAs.

**Table 3:** Host biological pathways targeted by helminth miRNAs according to predictions.

**Pathways**	**Helminth-host association**
**Common to all helminth-host associations**	
T cell receptor signaling, Ras signaling, Rap1 signaling, FoxO signaling, ErbB signaling	Asu/Ssc, Hco/Oar, Hpo/Mmu, Tmu/Mmu, Cel/Mmu, Cel/Ssc, Cel/Oar
**Other pathways of interest in associations involving parasitic species**	
Mucin type O-glycan biosynthesis	Asu/Ssc
Notch signaling pathway	Asu/Ssc, Hpo/Mmu
TGF-beta signaling pathway	Hco/Oar, Tmu/Mmu, Hpo/Tmu
mTOR signaling pathway	Asu/Ssc, Hco/Oar, Tmu/Mmu, Hpo/Mmu
Th17 cell differentiation	Asu/Ssc
**Pathways of interest targeted by species-specific miRNA seeds**	
AGE-RAGE signaling pathway	Asu/Ssc, Hco/Oar, Hpo/Tmu

*Selected significantly enriched host pathways are listed, according to adjusted p-value (p < 0.05), focusing on elements of the host immune system. Asu, A. suum; Hco, H. contortus; Hpo, H. polygyrus; Tmu, T. muris; Cel, C. elegans; Ssc, S. scrofa; Oar, O. aries; Mmu, M. musculus*.

We further stratified the results, focusing on the targets of species-unique miRNAs, based on seed sequences. Varying numbers of unique, species-specific miRNAs [*A. suum* = 8; *H. contortus* = 10; *H. polygyrus* = 26; *T. muris* = 32; *C. elegans* (target search vs. mouse genes) = 36] targeted varying numbers of KEGG pathways under the significance level (adj. *p* < 0.05) [*A. suum* = 42; *H. contortus* = 3; *H. polygyrus* = 25; *T. muris* = 54; *C. elegans* (target search vs. mouse genes) = 80]. All targeted the FoxO signaling pathway, via different genes or sites on the same gene, illustrating miRNA pleiotropy. Here too, pathways such as the MAPK, and TGF-β signaling pathways were enriched in most host-parasite associations (Supplementary Table S6). Interestingly, all parasites but *H. contortus* and *C. elegans*, targeted AGE-RAGE signaling, a pro-inflammatory pathway.

## Discussion

Despite methodological differences, our miRNA sequencing results are in agreement with those of Russell et al. (2018). Similarly, the proteomic data parallel those from the prior report at the functional level, and a large proportion of proteins are associated with membranous structures, which we expected from EV-rich preparations; both studies report many c-type lectins. On EV surfaces, lectins may mediate docking to recipient cells via proteoglycans (French et al., 2017). Interestingly, lectins were not observed on the surface of *F. hepatica* derived EVs, while molecules with peptidase activity, also abundant in our dataset, were highly represented (de la Torre-Escudero et al., 2019). Helminth EVs were reported to typically transport cytoskeletal proteins (e.g., actin), stress-related proteins (e.g., heat-shock proteins), along with Rab proteins and others (Mekonnen et al., 2018), which we also identified in the present work.

miRNA sequencing allowed the identification of 100 miRNAs from EV-enriched culture media. Whether this list results from sorting of specific miRNA species for export via EVs, showing higher proportions of 3′ uridylated isoforms, as shown in mammalian B cells and derived EVs (Koppers-Lalic et al., 2014), remains to be defined. The miRNA profile we obtained from EV-enriched processed culture media is fairly different from those from parasitic nematodes characterized so far, where the usually abundant miR-100 isoforms (miR-10-P2 reads according to MirGeneDB), bantam, mir-71, and mir-279, typically occupy the top ranks in abundance. A previous comparative study revealed that miR-100, miR-92, miR-279, and miR-137 (miRBase IDs) are hallmarks of parasitic nematode species, as they could not be identified in *C. elegans* (Wang et al., 2015b), suggesting their importance in parasitic processes. These miRNAs are also missing from our high-confidence list. *Let-7* strongly accumulates in the L4 stage and is required for the transition between larval to adult stages (Reinhart et al., 2000). miR-80 (cel-bantam-P2_3p) is a major metabolic regulator, broadly expressed in tissues of well-fed animals and down-regulated in the absence of food (Vora et al., 2013). The abundance of miR-80 in the EV-enriched fraction suggests that no starvation response had been activated during the short incubations. The MIR-216 family, comprising miRNAs with highly heterogenous miRBase annotations, appeared to be represented in parasitic species only; the literature mentioning miRNAs assigned to this family is very scarce. MIR-71 and MIR-54 were absent in the only clade I parasite and may reflect an evolutionary distance. BANTAM was missing in *H. contortus*; mir-5885 isoforms were, however, found to be homologous to *Drosophila* bantam (Marks et al., 2019). Similarly, five further miRNA families were found in all nematodes except *A. suum*. Although these differences may reflect phylogeny, life-cycle requirements, and/or host specificity, they might as well be the result of varying dataset sizes and completeness.

The first experimental confirmation of host manipulation by parasitic nematode miRNAs was obtained with the murine pathogen *H. polygyrus*, which releases EVs enriched with Argonaute proteins and miRNAs (Buck et al., 2014; Coakley et al., 2017). The nematode's miRNAs were shown to downregulate host *Il33r* and *Dusp1 in vitro*, leading to the suppression of the Th2 innate immune response to an allergen *in vivo* (Buck et al., 2014). This pioneer work was followed by several further reports on other helminths (Wang et al., 2015a; Zamanian et al., 2015; Fromm et al., 2017; Zheng et al., 2017; Eichenberger et al., 2018a,c; non-exhaustive list).

miRNAs are highly conserved through the animal kingdom. miRNA function is characterized by redundancy and pleiotropy, whereby the expression level of one transcript may be regulated by several miRNAs, and one miRNA binds to many targets, establishing a complex regulatory network. Differences in the miRNA profiles of *C. elegans* and parasites had no overt functional consequences resulting from the target search analysis. Narrowing down the search to targets of the top 20 most abundant miRNAs did not lead to substantial differences in targeted pathways. This is best explained by the fact that identical seed sequences are found in different miRNAs from different families: for instance, if the miR-100 isoforms are absent in *C. elegans*, the corresponding seed is found in miR-51 and others, abundantly represented in the nematode secretions. There is currently no consensus on the amounts of miRNAs required to observe a phenotype in the interaction between a host and a parasite, or in other words, whether a threshold for biological significance may exist. The overall low exosomal miRNA concentrations were even proposed to be biologically insignificant in natural settings (Chevillet et al., 2014).

The miRNA species, number, families, and seed sequences appeared to vary substantially across the examined reports and our current data. There are a number of elements that require caution while comparing miRNA datasets, currently. It is expected that not all organisms release equal amounts of EVs; it was shown previously that some nematode stages are more productive than others (Zamanian et al., 2015). The general experimental designs implemented for EV collection may not reflect the natural *in vivo* situation, inducing stress on nematodes, which may have consequences on EV release and their cargo. Here, we attempted a comparison between miRNA species from juvenile non-parasitic hermaphrodites and adult dioecious parasites; however, it is known that nematode stages release different miRNA profiles (Tritten et al., 2016; Hansen et al., 2019). Finally, the miRNA library preparation and sequencing strategies are known to impact on the data, and there are no recommendations for standardized reporting to date.

Comparing free-living with parasitic species can be used as an approach to uncover the molecular basis of nematode parasitism. The underlying rationale is that parasitic nematodes have evolved from free-living ancestors, and therefore, parasites will have adapted existing ancestral traits and evolved new ones, which together underlie the parasitic lifestyle (Viney, 2018). By doing so, the most relevant comparisons are those taking into account the multiple evolutions of nematode parasitism, reflected in the nematode phylogeny (Blaxter et al., 1998). Ideally, the most appropriate and powerful phylogenomic comparisons are those that compare taxa within the clades, rather than between clades (Viney, 2018). The limited number of studies providing a characterization of EVs and sufficient miRNA information including sequence data does not allow such a design currently. In our current study, involving by chance only gastrointestinal pathogens, only *H. contortus, H. polygyrus*, and *C. elegans* belong to the same clade (V). They shared three seed sequences *a priori* absent in the other organisms.

Taking all reported high confidence miRNAs into account, we noted that, regardless of the size of the dataset, a large proportion of seed sequences was unique to each species (or dataset). We assessed whether nematode miRNA seed identity to host miRNAs may constitute an advantageous trait associated with parasitism, bringing the capacity to subvert to existing host regulatory pathways. The proportion of identical seed sequences was not higher in parasite-host comparisons than in those between parasites and *C. elegans*. We observed that the parasite-secreted miRNA fractions shared more often seed sequences with host miRNAs than those that are not found in the extracellular milieu. Overall, this suggests that miRNA release by parasites could constitute an evolved mechanism of host modulation, in an extent that we have barely started to recognize. Regulating conserved host endogenous sites could be effective for two reasons: (i) the context of these sites already permits functional repression, and (ii) these sites cannot mutate to prevent pathogen regulation without modifying/compromising the host physiology (Claycomb et al., 2017). The likely mechanisms and implications of co-evolution between host mRNA and parasite miRNA have been addressed by Claycomb et al. (2017).

Target search and pathway analysis based on whole miRNA repertoires did not allow clustering across lifestyles or clades. *C. elegans* miRNA targets among mammalian genes revealed the same most highly enriched pathways within broad immune functions. This is likely due to the redundant and pleiotropic properties of miRNA-driven gene regulation. According to predictions, aspects in T cell differentiation and proliferation were repeatedly among impaired pathways. Similarly, the PI3K/Akt signaling is central in T cell development (among other cells; Juntilla and Koretzky, 2008). In lymphocytes, Notch is essential for specifying T cell fate and for promoting early T cell differentiation (Laky and Fowlkes, 2008). The importance of mTOR has been shown in DC-mediated T helper cell differentiation (Salmond and Zamoyska, 2011; Hussaarts et al., 2013). Interestingly, *A. suum* miRNAs were predicted to affect the mucin type O-glycan biosynthesis pathway. Increased mucus production represents a key protective mechanism against gastrointestinal nematodes, to facilitate expulsion of the pathogens (Sharpe et al., 2018). Defective mucin type O-glycan biosynthesis leads to disruption of gut homeostasis and to the re-shaping of the whole gut microbial community (Bergstrom and Xia, 2013). In *T. muris* infections, transforming-growth factor beta (TGF-β) seems to be pivotal in regulating responses to the worm: when CD4+ T cells have reduced ability to respond to TGF-β (due to the expression of truncated version of TGF-β receptor II), mice harbored higher worm burdens and down-regulated levels of Th2 cytokines (Veldhoen et al., 2008; Klementowicz et al., 2012). The AGE-RAGE signaling pathway was the only convergent enrichment result common to species-specific miRNA seeds from three parasitic species, but not *C. elegans*. The influence of dietary advanced glycation end-products (AGE) and its receptor RAGE in an immune context is known in other infectious diseases (Traoré et al., 2016). RAGE is a pattern recognition receptor expressed by B and T cells, as well as dendritic cells. Its expression and response to released RAGE ligands have been observed in human eosinophils Curran and Bertics (2011), a hallmark of immune responses to helminth infections. Upon activation, it is involved in immediate inflammatory response, and a shift toward lymphocyte Th1 differentiation (Kierdorf and Fritz, 2013; Traoré et al., 2016).

Due to inherent bias in the length, conservation, and nucleotide composition of 3′UTR gene portions, target prediction tools suffer some imperfection that can result in prediction inaccuracy. Therefore, computational target predictions require experimental validation to provide more conclusive answers. Our efforts were restricted to seed sequence length of 7 nucleotides for practical reasons; in reality, these may range from 6 to 8 nucleotides.

There is as yet no general mechanistic framework describing how RNA trafficking is programmed; whether and how EV cargo is targeted, what needs to be co-delivered, and how a given miRNA may be integrated into a functional pathway in a recipient cell remain unknown (Claycomb et al., 2017). Cross-species and cross-kingdom communication relying on RNA is a young research field that holds promise for the design of novel diagnostic and disease control strategies. Based on presence or absence of miRNA candidates in each dataset, this approach does not address the quantitative dynamics of sequences with immunomodulatory properties. Here, we propose a simplistic multi-species comparative approach to identify clustering patterns among miRNA sequence-based regulatory networks across organisms. With increasing parasite miRNA collections with improved data depth and quality, this work will certainly find even more resonance and significance in a near future.

## Data Availability Statement

The miRNA sequencing data have been deposited to GEO (NCBI) with the dataset GSE144289. The mass spectrometry proteomics data have been deposited to the ProteomeXchange Consortium via the PRIDE (Vizcaíno et al., 2014) partner repository with the dataset PXD017352. The analysis codes are available upon request to the corresponding author.

## Author Contributions

TD and LT designed the study and wrote the manuscript. TD, JS, and LT carried out the experiments and analyzed the data. RK and LM assisted in processing the data. All authors read and approved the submitted version.

## Conflict of Interest

The authors declare that the research was conducted in the absence of any commercial or financial relationships that could be construed as a potential conflict of interest.
