# Supplementary material for: Extracellular Vesicle-Contained microRNA of C. elegans as a Tool to Decipher the Molecular Basis of Nematode Parasitism
Source: Front Cell Infect Microbiol. 2020 May 25;10:217. doi: 10.3389/fcimb.2020.00217 (PMC7261840; doi:10.3389/fcimb.2020.00217)
Supplement: Supplementary file 7 [file Data_Sheet_1.PDF]

miRNA sequencing – quality control and read statistics

1) **RNA sample - Bioanalyzer**

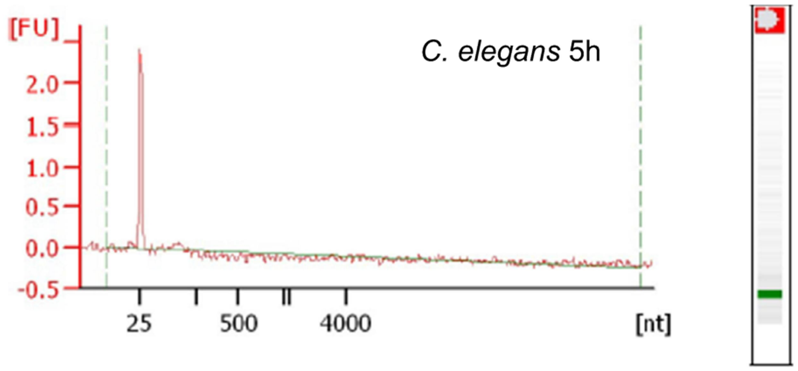

Overall results for *C. elegans* 5h

RNA Area:	1.2
RNA Concentration:	6 pg/µl
rRNA Ratio [28s / 18s]:	0.0
RNA Integrity Number (RIN):	N/A (B.02.08)
Result Flagging Color:	
Result Flagging Label:	RIN N/A

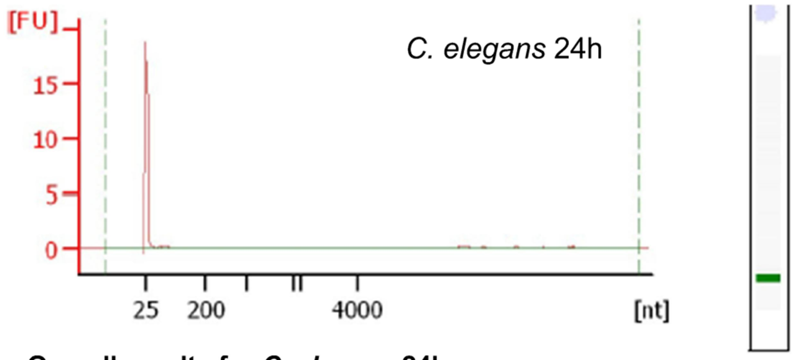

Overall results for *C. elegans* 24h

RNA Area:	2.7
RNA Concentration:	13 pg/µl
rRNA Ratio [28s / 18s]:	0.0
RNA Integrity Number (RIN):	1 (B.02.08)
Result Flagging Color:	
Result Flagging Label:	RIN:1

2) cDNA library – Bioanalyzer

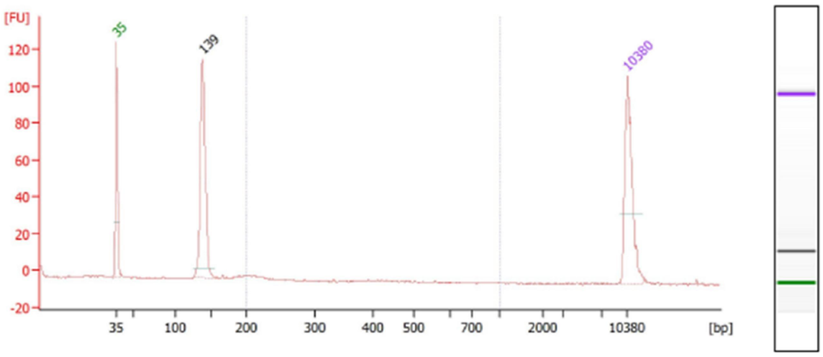

Overall results for *C. elegans* 5h

Number of peaks found:	1	Corr. Area 1:	6.8
Noise:	0.3		

Peak table

Peak		Size [bp]	Conc. [pg/µl]	Molarity [pmol/l]	Observations
1	◀	35	125.00	5,411.3	Lower Marker
2		139	227.13	2,484.3	
3	▶	10,380	75.00	10.9	Upper Marker

Region table

From [bp]	To [bp]	Corr. Area	% of Total	Average Size [bp]	Size distribution in CV [%]	Conc. [pg/µl]	Molarity [pmol/l]	Color
200	1,000	6.8	3	235	28.5	7.20	49.7	■

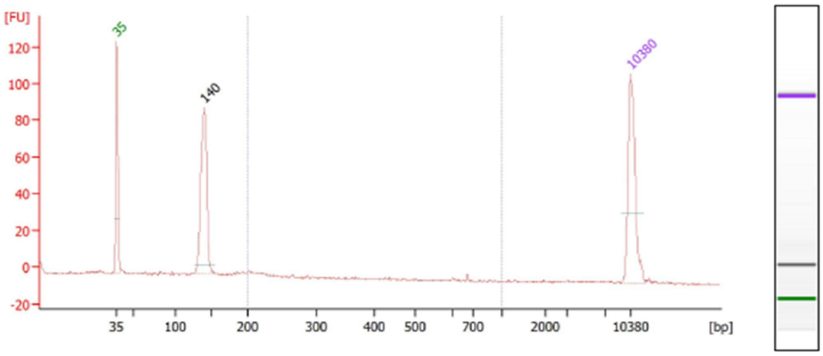

Overall results for *C. elegans* 24h

Number of peaks found:	1	Corr. Area 1:	14.5
Noise:	0.4		

Peak table

Peak		Size [bp]	Conc. [pg/µl]	Molarity [pmol/l]	Observations
1	◀	35	125.00	5,411.3	Lower Marker
2		140	212.74	2,302.4	
3	▶	10,380	75.00	10.9	Upper Marker

Region table

From [bp]	To [bp]	Corr. Area	% of Total	Average Size [bp]	Size distribution in CV [%]	Conc. [pg/µl]	Molarity [pmol/l]	Color
200	1,000	14.5	6	302	53.2	15.60	97.4	■

3) Read statistics

miRNA sequencing – read statistics

		C. elegans 5h				*C. elegans* 24h			
**lib**	**Type**	**Total**	**% of total**	**Unique**	**% of unique**	**Total**	**% of total**	**Unique**	**% of unique**
**Raw reads**	NA	13733359	100	531323	100	11198407	100	432627	100
**3ADT&length filter**	Sequence type	4208636	30.65	353554	66.54	872979	7.8	244894	56.61
**Junk reads**	Sequence type	35766	0.26	1845	0.35	7078	0.06	1174	0.27
**Rfam**	RNA class	1553173	11.31	8919	1.68	211355	1.89	4222	0.98
**mRNA**	RNA class	92831	0.68	2678	0.5	39516	0.35	617	0.14
**Repeats**	RNA class	9047	0.07	183	0.03	5634	0.05	126	0.03
**valid reads**	Sequence type	7851394	57.17	164337	30.93	10066020	89.89	181707	42
**rRNA**	RNA class	777015	5.66	5932	1.12	139438	1.25	2699	0.62
**tRNA**	RNA class	105366	0.77	1662	0.31	49690	0.44	999	0.23
**snoRNA**	RNA class	3860	0.03	171	0.03	2595	0.02	102	0.02
**snRNA**	RNA class	3648	0.03	83	0.02	2768	0.02	66	0.02
**other Rfam RNA**	RNA class	663284	4.83	1071	0.2	16864	0.15	356	0.08
