# Supplementary material for: Extracellular Vesicle-Contained microRNA of C. elegans as a Tool to Decipher the Molecular Basis of Nematode Parasitism
Source: Front Cell Infect Microbiol. 2020 May 25;10:217. doi: 10.3389/fcimb.2020.00217 (PMC7261840; doi:10.3389/fcimb.2020.00217)
Supplement: Supplementary file 8 [file Data_Sheet_2.pdf]

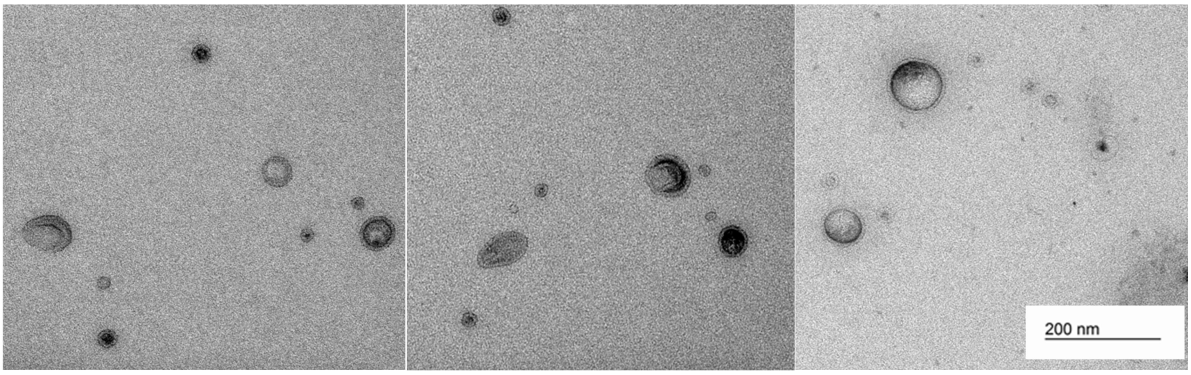

Supplementary Figure 1. Characterization of extracellular vesicles by transmission electron microscopy. Representative images from three independent EV isolations. The typical deflated ball shape of exosomes can be observed.

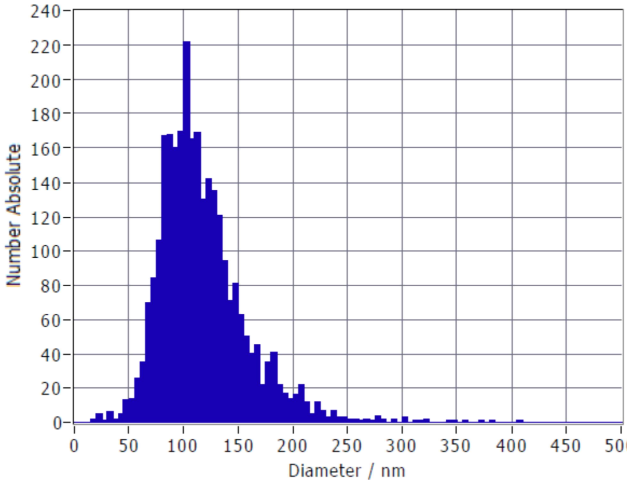

Supplementary Figure 2. Nanoparticle tracking analysis of EVs in *C. elegans* processed culture media. Size distribution of observed EVs in a diluted sample collected after 24 h incubation.
